# P-2107. Diagnostic accuracy of Nucleic Acid Amplification Tests for detection of *Mycobacterium leprae* in suspected leprosy patients from northern region of India

**DOI:** 10.1093/ofid/ofae631.2263

**Published:** 2025-01-29

**Authors:** Rakesh Yadav, Gurmeet Saini, Manjot Kaur, Tarun Narang, Sunil Dogra, Sunil Sethi

**Affiliations:** PGIMER, Chandigarh, Chandigarh, Chandigarh, India; PGIMER, Chandigarh, Chandigarh, Chandigarh, India; PGIMER, Chandigarh, Chandigarh, Chandigarh, India; PGIMER, Chandigarh, Chandigarh, Chandigarh, India; PGIMER, Chandigarh, Chandigarh, Chandigarh, India; PGIMER, Chandigarh, Chandigarh, Chandigarh, India

## Abstract

**Background:**

Leprosy is a chronic infectious disease, still remains a significant health problem in several parts of the world. A rapid and accurate diagnosis of the disease is essential for better management of Leprosy cases. In this study we compared three molecular methods i.e. PCR, Real TIME PCR and Loop-mediated isothermal amplification (LAMP) assay for detection of mycobacterium leprae.

**Methods:**

A total of 114 samples were taken from 92 clinically leprosy suspected patients and contacts. DNA was extracted from the samples and molecular assays i.e. PCR, Real TIME PCR and LAMP was performed in parallel. Diagnostic accuracy of each test and Cohen's kappa coefficient was calculated using medcal online software.

**Results:**

Among the 114 samples tested, the M leprae was detected in 35.09%(40/114), 34.2% (39/114) and 32.4%(37/114) by LAMP, conventional PCR, and real-time PCR, respectively. The overall agreement between conventional PCR and LAMP for detection of M leprae was 97.3% with a Cohen's kappa coefficient of 0.94, indicating perfect agreement. Similarly, the agreement between real-time PCR and LAMP for detection of M leprae was 97.3% with a Cohen's kappa coefficient of 0.94. However, the overall diagnostic accuracy of PCR, Real Time PCR and LAMP for detection of M leprae was 83.2%, 81.2% and 81.2%, respectively.

**Conclusion:**

All molecular assays i.e. PCR, Real TIME PCR and LAMP had shown a similar diagnostic accuracy for detection of mycobacterium leprae.

**Disclosures:**

All Authors: No reported disclosures

